# Daily patterns and prevalence of behavioral and psychological symptoms of dementia in Swedish nursing homes

**DOI:** 10.1186/s12877-026-08537-w

**Published:** 2026-10-29

**Authors:** Johannes Malm, Charlotta Nilsen, Ingemar Kåreholt

**Affiliations:** 1 Institute of Gerontology, School of Health and Welfare, Jönköping University, Jönköping, Sweden; 2 Department of Health, Blekinge Institute of Technology, Karlskrona, Sweden; 3 Aging Research Center (ARC), Department of Neurobiology, Care Sciences and Society (NVS), Karolinska Institutet/Stockholm University, Stockholm, Sweden; 4 The Stress Research Institute, Department of Psychology, Stockholm University, Stockholm, Sweden; 5 Division of Clinical Geriatrics, Department of Neurobiology, Care Sciences and Society (NVS), Karolinska Institutet, Stockholm, Sweden

**Keywords:** BPSD, Dementia, Nursing homes, Daily observations, Longitudinal studies, Prevalence, Person-centered care

## Abstract

**Background:**

Behavioral and psychological symptoms of dementia (BPSD) are among the principal observable expressions of psychological distress and unmet need in nursing home residents who can no longer reliably verbalize internal experiences, and recognizing their day-to-day variation underpins timely, person-centered care. Yet BPSD fluctuate across days, shifts, and contexts, while established instruments such as the Neuropsychiatric Inventory Nursing Home version (NPI-NH) assess them retrospectively over the preceding weeks or months. This mismatch leaves basic descriptive questions about the day-to-day symptom landscape in routine care unanswered. This study describes the prevalence, severity, co-occurrence, between-unit variation, and shift and weekday patterns of BPSD documented through structured daily registrations in nursing home care.

**Methods:**

Descriptive longitudinal observational study of 230 individuals with dementia in six nursing homes across six Swedish municipalities, followed for a median observation period of 270 days (interquartile range 120 to 446). Care staff used a digital tool to rate the severity (0–3) of twelve common BPSD, adapted from the NPI-NH, at the end of every work shift, yielding over one million symptom registrations. Analyses were descriptive and person-weighted, giving each individual equal weight regardless of observation length.

**Results:**

Cumulative symptom prevalence (at any point during observation) ranged from 90% (agitation) to 70% (euphoria). Each symptom’s mean daily prevalence was much lower (4–18%), and symptoms were predominantly of low severity. At least one symptom was recorded on a median of 43.5% of registered days, varying widely between individuals. Clinically related symptoms tended to occur together, consistent with established NPI-NH domain structures, and the mean number of symptoms per day varied more than twofold across the six units. Symptoms were most frequent on evening shifts and least frequent at night, except sleep disturbances, with little variation across weekdays. Just under half of all possible shift registrations were completed.

**Conclusions:**

Daily registrations describe an intermittent and heterogeneous symptom landscape at a resolution that periodic assessments are not designed to provide. These findings provide an empirical baseline for future multilevel analyses and support BPSD monitoring that covers both individual symptoms and the daily number of symptoms in nursing homes.

**Clinical trial number:**

Not applicable.

**Supplementary information:**

The online version contains supplementary material available at https://doi.org/10.1186/s12877-026-08537-w.

## Background

Dementia is a progressive neurocognitive condition affecting a large and growing proportion of older adults worldwide. Current estimates suggest that the global number of people living with dementia will rise from approximately 57 million in 2019 to over 150 million by 2050 [1]. Beyond cognitive decline, up to 90% of individuals with dementia develop behavioral and psychological symptoms (BPSD) at some point in the disease course [2, 3]. These symptoms, which include agitation, anxiety, depression, hallucinations, apathy, and sleep disturbances, are heterogeneous, fluctuating, and often distressing for the individuals concerned [4, 5]. For people with dementia who can no longer reliably verbalize internal experiences, BPSD are among the principal observable expressions of psychological distress and unmet need, and their presence is closely linked to reduced quality of life and increased caregiver burden [5, 6].

In nursing homes, where many residents with dementia live their final years, recognizing and responding to this variation is central to person-centered care [7, 8]. Effective non-pharmacological interventions, such as environmental adjustment, individualized activities, and communication strategies, depend on understanding the context in which an individual’s symptoms occur [9], including how they vary across days and shifts. The same information supports the everyday work of care: noticing early signs of symptom worsening before they intensify, judging whether a recent change in environment, activity, or treatment is having its intended effect, and sustaining continuity of information across shift handovers [10, 11]. Underpinning each of these is a shared, day-to-day picture of the resident, one that care teams, clinicians, and families can refer to and act from. BPSD vary substantially in their prevalence, intensity, and distress [12], yet the systems used to monitor them in routine practice rarely capture this variation [13].

The most widely used framework for assessing BPSD is the Neuropsychiatric Inventory and its nursing home version (NPI-NH) [14, 15], with translations spanning approximately 40 languages and broad international uptake [16]. The instrument is, however, administered retrospectively, typically covering the preceding month [17], and national initiatives such as the Swedish BPSD Registry conduct follow-up registrations every six to twelve months [18]. This creates a structural mismatch between phenomenon and measurement: BPSD vary across days, shifts, and contexts, yet prevailing assessment practices, typically the NPI-NH, summarize symptoms over a recall period and depend on staff recall, limiting the ability to detect clinically meaningful day-to-day variation.

This mismatch between how BPSD behave and how they are measured leaves fundamental descriptive questions about routine nursing home care unanswered. Recent work has demonstrated the value of intensive longitudinal data for identifying BPSD subsyndromes and within-person symptom clusters [19], but to our knowledge no study has provided a comprehensive descriptive account of BPSD patterns derived from structured daily registrations applying the NPI-NH symptom framework. A digital tool developed through participatory design with nursing home staff and relatives in Sweden [20] addresses this gap by adapting the NPI-NH for shift-level documentation by direct care staff [21]. An initial mixed-methods pilot in four nursing homes indicated that this registration routine could be integrated into everyday dementia care practice [21]. Severity is rated at the end of each work shift on a four-level scale adapted from the NPI-NH (levels 1 to 3 retaining its severity anchors, with 0 denoting absence), producing a longitudinal, shift-level record of symptom expression at the timescale at which symptoms actually fluctuate.

The multi-site nature of these data also opens a second descriptive question. Earlier cross-sectional work has shown that BPSD prevalence varies between dementia care units [22], and the present study describes whether, and how much, such variation appears in continuous, prospectively collected daily records.

The aim of this study was to provide a comprehensive descriptive account of BPSD as documented through structured daily registrations in Swedish nursing home care. Specifically, the study describes:

1. the prevalence and severity distribution of individual symptoms;
2. patterns of symptom co-occurrence;
3. variation in symptom burden across care units; and
4. symptom patterns across shifts and weekdays.

## Methods

This is a descriptive longitudinal observational study based on data collected through a digital documentation tool across six nursing homes in Sweden. The study follows the STROBE guidelines for reporting observational studies [23]. The completed checklist is provided in Additional file 1 (Table S1).

### Setting and data source

Data were collected between January 2024 and December 2025 in six nursing homes, each represented by one care unit (hereafter units; U1–U6) comprising one to six wards (18 in total; W1–W18), in six Swedish municipalities (Municipalities A–F). Units started using the tool at different dates between January and October 2024 (Additional file 1, Table S2). Two of the six units (U1 and U3) had previously taken part in the mixed-methods pilot of the tool [21]. Of the remaining four, two contacted the research team after learning about the pilot, and two were recruited through professional networks in health and social care for older people. The units included both municipal and privately operated facilities. All wards were specialized dementia care wards providing 24-hour residential care for individuals with dementia.

The tool, referred to here as Daily-BPSD, is a purpose-built digital instrument designed for systematic daily documentation of BPSD in nursing home settings. The tool was developed through participatory design with nursing home staff and relatives [20] and adapted from the Neuropsychiatric Inventory – Nursing Home version (NPI-NH) [15]. Each symptom is rated on a four-level scale in which levels 1 to 3 retain the standard NPI-NH severity anchors, 1 (mild), 2 (moderate), and 3 (severe), while level 0 denotes absence. Unlike the standard NPI-NH, no separate frequency dimension is recorded. At each shift, all twelve domains are rated for every resident, so a rating of 0 reflects an active judgment that the symptom was absent during that shift rather than a missing entry, and a shift for which no form was completed is treated as a missing shift registration (see Statistical approach) rather than as absence of symptoms. To support consistent interpretation, each domain is accompanied by an on-screen definition and behavioral examples that staff can consult at the point of registration. Because a shift registration can be completed up to three times daily, the tool yields a record of symptom expression at shift-level resolution.

Registrations are carried out by direct care staff (i.e., nursing assistants and assistant nurses providing hands-on daily care) at the end of each work shift: night, morning, and evening. In addition to the twelve NPI-NH symptom domains, the tool includes individually selected variables (e.g., food intake, sleep observations, use of pro re nata (PRN) medication, i.e., as-needed administration) chosen by care staff at each unit. Only the standardized NPI-NH symptom items are analyzed in the present study. Throughout this article, a “registration” (or “item-level registration”) refers to a single severity rating recorded for one NPI-NH symptom domain for one resident at one shift, rather than to a staff login or form submission. Reported registration totals therefore reflect the number of item-level rating events across the twelve NPI-NH domains. A “shift registration” refers to one completed NPI-NH form for one resident at one shift (up to three per resident per day, one per shift).

Data processing and analysis were performed in Python (version 3.12; [24]) using pandas (version 3.0; [25]), NumPy (version 2.4; [26]), and matplotlib (version 3.10; [27]).

### Study population

The study population was drawn from residents of dementia care wards in the participating nursing homes during the data collection period. Admission to these wards requires a dementia diagnosis under Swedish elder-care practice, although the type and severity of diagnosis were neither verified nor used as inclusion or exclusion criteria in this study. Residents were registered in the tool by care staff as part of the staff’s daily care documentation, except residents whose representatives had objected to participation (see Ethics and data access). The cohort was dynamic. When a resident moved out or died, newly admitted residents were incorporated into the registrations, resulting in varying observation periods across individuals.

All 268 residents with Daily-BPSD registrations were considered for analysis. To restrict the analyses to periods of regular use, each resident’s observation period was defined by an active-use criterion based on consecutive 30-day blocks counted from the first registration date. The observation period for each resident began with the first 30-day period in which the tool was used on at least 15 days and ended with the last such period. Shorter gaps in between were kept. Use on at least 15 of 30 days corresponds to use on at least every other day, which we considered a reasonable indication of regular engagement with a daily instrument (the effect of alternative thresholds is reported in Additional file 1, Table S3). The criterion thus concerns the selection of data for analysis rather than enrollment in the study. For 38 residents, registration never reached this level in any 30-day period. These residents, for whom the tool was never used systematically enough to support the daily-resolution analyses that are the focus of this study, were not analyzed. Among the remaining 230 residents, registrations outside the observation period were removed for 183 (54,649 registrations).

**Fig. 1:**
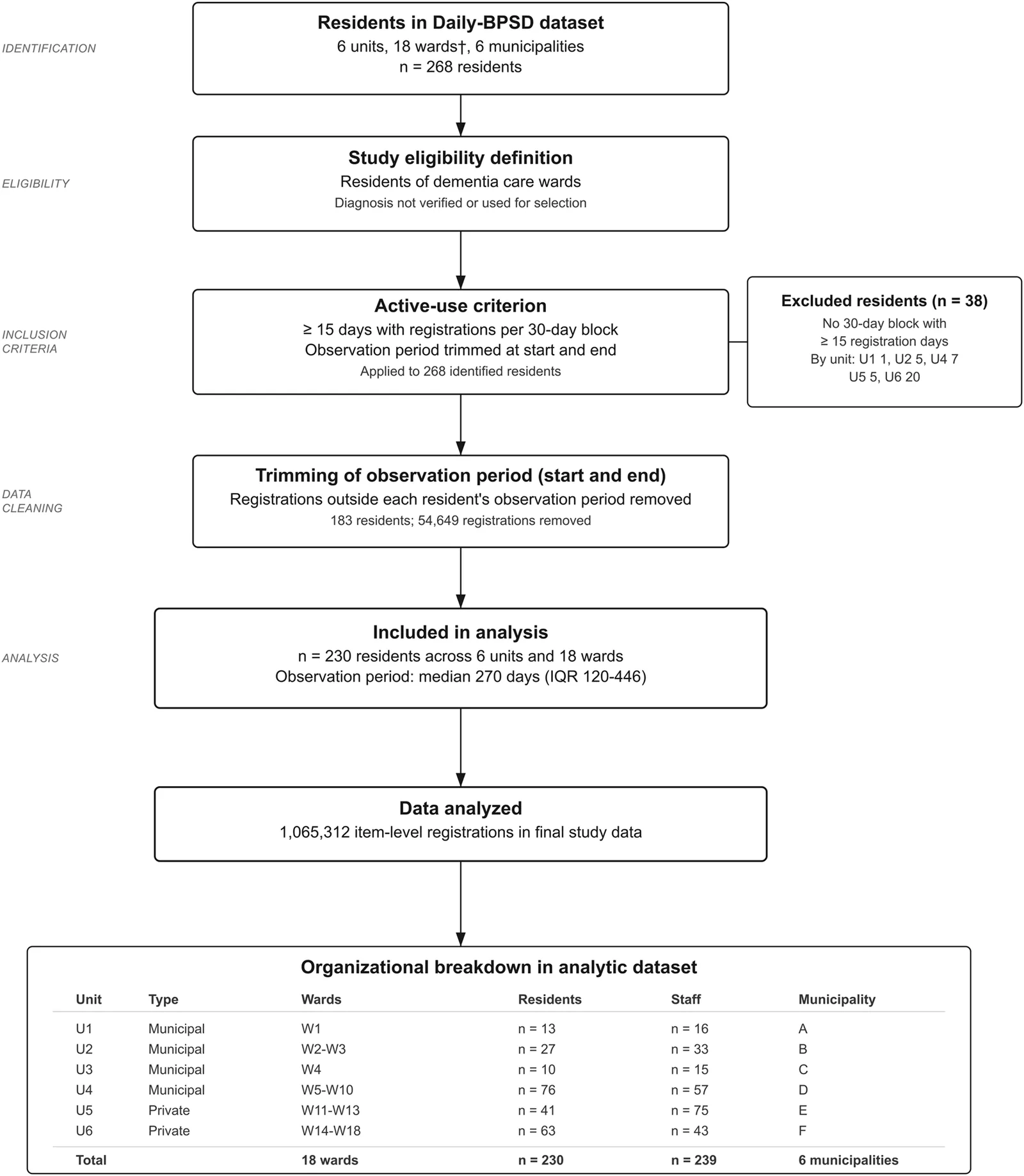
Flow diagram of participant selection. Of 268 residents with Daily-BPSD registrations, 230 met the active-use criterion and were included in the analyses. †Registrations were made on 21 wards; three of these had only residents who did not meet the criterion, so 18 wards contributed analyzed data. Staff is the number of staff members who made registrations for the included residents

The final sample comprised 230 individuals (P1–P230) across 18 wards and 6 units, with a median observation period of 270 days (interquartile range [IQR] 120–446), yielding 1,065,312 item-level registrations across the twelve NPI-NH domains. The complete selection process is illustrated in Fig. 1.

### Variables and data structure

The data are organized in a six-level hierarchical structure: (1) item registration, (2) shift (night, morning, or evening), (3) day, (4) resident, (5) ward, and (6) unit. This nested structure reflects the dependencies inherent in the data. Registrations within the same shift, day, individual, and ward are not independent observations, which does not bias the descriptive estimates reported here but must be accounted for in any future inferential analysis.

The present study analyzes the twelve NPI-NH symptom domains: delusions, hallucinations, agitation, depression, anxiety, euphoria/elation, apathy, disinhibition, irritability/emotional lability, motor restlessness, sleep disturbances, and appetite and eating disturbances. Each symptom is rated on the four-level severity scale (0–3) described above. A symptom is classified as present when the registered severity is greater than zero.

### Statistical approach

All analyses are descriptive. To give each person the same weight in the analyses, and to avoid individuals with more registered days dominating aggregated estimates, person-weighted means are used throughout. Each measure is first computed per individual, and the resulting individual-level values are then averaged across the sample. For example, a resident observed for 30 days contributes as much to each estimate as a resident observed for 300 days.

Symptom prevalence is reported at two levels: cumulative prevalence (the proportion of individuals with at least one recorded occurrence over the entire observation period) and mean daily prevalence (the person-weighted mean proportion of registered days on which a symptom was present). Daily prevalence was calculated with registered days (days with at least one shift registration) as the denominator. Days without any shift registration were excluded rather than treated as symptom-free. Day-level severity was defined as the maximum severity recorded across that day’s shifts. Night shifts, which span midnight, were assigned to the date on which they ended, which is also the date of registration. A day therefore consisted of the night shift ending that morning, followed by the morning and evening shifts. Severity is reported as the person-weighted percentage of registered days on which each symptom was recorded at level 1 (mild), 2 (moderate), and 3 (severe). The overall severity distribution was computed per individual as the share of days on which a symptom was recorded, across all symptoms, at each severity level (the highest rating across that day’s shifts) and then averaged across individuals.

Because the number of registered shifts varied between days, daily prevalence was also computed for days with all three shifts registered, and shift-level prevalence as the person-weighted proportion of shift registrations at each shift in which the symptom was recorded, together with the proportion of shift registrations with at least one symptom (Additional file 1, Table S4). To examine whether registration depended on symptom occurrence, the proportion of shift registrations with at least one symptom was compared within residents and within the same shift between days with one and with all three shifts registered (173 to 180 residents per shift). The between-unit comparison, the co-occurrence analysis, and the Gini coefficient were also repeated on days with all three shifts, among the 185 residents with at least one such day (10 to 65 per unit; for phi, at least 77 residents per pair, median 105). Phi was additionally computed with single shift registrations rather than days as the unit (at least 147 residents per pair). The relation between residents’ mean number of shifts per registered day and their mean daily symptom count (*n* = 230) was described with a Spearman rank correlation, without hypothesis testing. Weekday patterns were described as the person-weighted proportion of registered days with any symptom, and the mean number of registered shifts per day, by weekday.

Daily symptom burden is defined as the number of unique symptoms with a registered severity greater than zero per individual per day. The distribution of burden categories (0, 1, 2, or 3 or more symptoms) is reported as person-weighted proportions. The concentration of burden across individuals is described by the share of the total burden (the sum of residents’ mean daily symptom counts) accounted for by the 20% of individuals with the highest mean daily symptom count and by the Gini coefficient, which ranges from 0 (equal distribution) to 1 (all burden in one individual).

Pairwise symptom co-occurrence is quantified using phi coefficients. The phi coefficient is a measure of association between two binary variables [28]. As elsewhere in the analyses, each resident was given equal weight: phi was computed for each resident from that resident’s daily present/absent data and then averaged across residents, rather than calculated from all residents’ days combined. The mean for each pair is taken over residents who showed variation in both symptoms (at least 147 of 230 residents for every pair). A phi of zero indicates that two symptoms co-occur no more often than expected by chance given their marginal frequencies, and higher values indicate stronger co-occurrence. Between-unit variation is described by comparing symptom-specific daily prevalence and the mean daily symptom count across the six units.

Registration completeness is examined across shifts, units, and time periods. Day-level completeness is the proportion of observation days (calendar days within each resident’s observation period) with at least one shift registration. Shift-level completeness is the proportion of possible shift registrations, three per observation day as the application affords, that were completed. All analyses are based on observed registrations only. All figures and tables use the study coding described above (U1–U6, W1–W18, P1–P230, Municipalities A–F).

### Ethics and data access

The study was approved by the Swedish Ethical Review Authority (Dnr 2022-04509-01) subject to conditions reflecting the cognitive vulnerability of the resident population. Because residents could not reasonably be expected to assimilate written study information themselves, the Authority directed that the information be addressed to a next of kin, legal guardian, or trustee, who was to consult with the resident. Under the Swedish Ethical Review Act (SFS 2003:460, Sects.  20 to 22), a representative cannot consent on a research participant’s behalf but may, after such consultation, object to participation. Participation therefore rested on non-objection rather than on consent. The information for representatives stated that they could object to the resident’s participation, or withdraw it at any time, by informing the staff, for example if there was reason to believe that the resident did not wish to take part. To minimize the research team’s handling of personal data, the information forms were distributed to the participating units and handled locally by the unit managers, who were responsible for ensuring that representatives had been informed and given the opportunity to object. No documentation from the non-objection procedure concerning individual residents was transferred to the research team, which was therefore not in a position to verify individual cases. Under this procedure, residents whose representatives objected were not registered in the tool. If a representative later withdrew the resident, the unit manager was to report this to the research team, identifying the resident only by the code used in the tool, and all previously registered data on the resident would then be deleted from the system if requested. No withdrawals were reported. The daily symptom registrations were embedded in documentation that care staff would otherwise perform during routine care, so participation required no additional procedure involving the residents. Use of the application required personal staff login to preserve accountability, in line with standard clinical documentation practice. Upon export to the research dataset, staff identifiers were replaced with study codes. The legal basis for processing personal data was a task carried out in the public interest (EU GDPR Art. 6(1)(e)). Results are presented only in aggregated form, and units, wards, and individuals are referred to by study codes.

## Results

The results are organized as follows: study population characteristics and data completeness are presented first, followed by symptom prevalence and severity, daily symptom burden, co-occurrence patterns, between-unit variation, and shift and weekday patterns, and finally the sensitivity of the day-level results to the number of registered shifts per day.

### Study population and data completeness

Table 1 summarizes the study population. The final sample comprised 230 individuals (123 female, 53.5%; 107 male, 46.5%) across 18 wards and 6 care units (4 municipal, 2 private), with a median age of 84 years (IQR 80–88). Median observation period was 270 days (IQR 120–446), yielding 1,065,312 item-level registrations in total. Sex was obtained from administrative care records. Gender identity was not recorded. Compared with the included residents, the 38 excluded residents were of similar age (median 85.5 vs. 84 years) but had few registered days (median 8.5) and a lower proportion of registered days with any symptom (median 16.9% vs. 43.5%). The number of ward residents who were never registered in the tool could not be determined, as occupancy data were not available. The characteristics of each unit are given in Additional file 1, Table S2.

**Table 1:** Study characteristics

Measure	Total
**Setting and sample**	
Care units, n (municipal/private)	6 (4/2)
Residents per ward, median (IQR)	12.5 (10–15)
**Demographics**	
Female, n (%)	123 (53.5)
Male, n (%)	107 (46.5)
Age at first registration, years, median (IQR)	84 (80–88)
**Observation period**	
Observation period, days, median (IQR)	270 (120–446)
Observation period, days, min–max	24–719
Registered days, median (IQR)	194 (99–365)
**Registration volume**	
Total registrations, n	1,065,312
Registrations per resident, median (IQR)	3,974 (1,818–6,994)
Registrations per resident, min–max	179–17,202
Registrations per observation day, median (IQR)	15.6 (12.1–21.0)

Note. *N* = 230 residents. Registrations refer to item-level symptom ratings (one NPI-NH domain, one resident, one shift; see Methods). Percentages are computed within the full study population

**Fig. 2:**
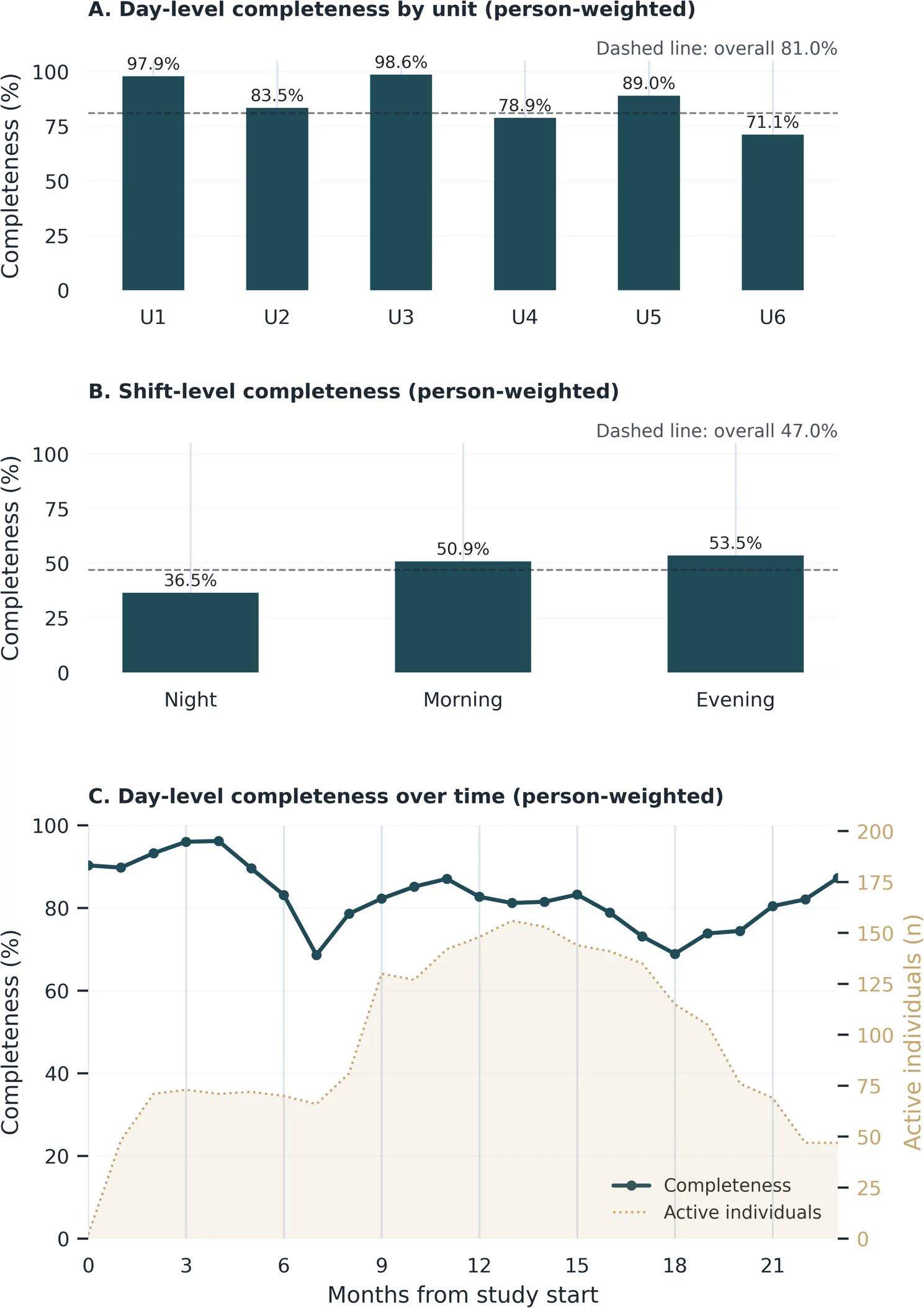
Registration completeness across units, shifts, and time. (A) Person-weighted day-level completeness by care unit. (B) Person-weighted shift-level completeness (the proportion of possible shift registrations that were completed) by shift. (C) Person-weighted day-level completeness over time (solid line, left axis); the shaded band and dotted line show the number of active individuals contributing data in each month (right axis). Dashed horizontal lines in panels A and B mark the overall mean

On average, at least one shift was registered on 81.0% of each resident’s observation days (person-weighted day-level completeness; Fig. 2, panel A). Completeness varied across units, ranging from 71.1% (U6) to 98.6% (U3). Shift-level completeness was 47.0% overall, ranging from 36.5% for night shifts to 50.9% for morning and 53.5% for evening shifts. In other words, just under half of all possible shift registrations were completed (Fig. 2, panel B). Every registered day had at least one shift registered by definition. Of these days, 53.5% had at least two shifts and 14.9% all three. Completeness was generally stable over time but showed recurring dips during summer months (Fig. 2, panel C).

### Symptom prevalence, severity, and daily burden

Over the whole observation period, each symptom was recorded at least once in most residents, from 70.4% of residents for euphoria to 90.4% for agitation (cumulative prevalence; Table 2). On any given day, however, a symptom was recorded far less often: the mean proportion of registered days with the symptom ranged from 4.3% (euphoria) to 17.6% (motor restlessness). Motor restlessness, agitation, and anxiety were the three most frequently occurring symptoms on a daily basis. Registered severity was predominantly mild. When a symptom was recorded on a day, its severity (the highest rating across that day’s shifts) was level 1 on 61.0% of such days, level 2 on 26.0%, and level 3 on 13.0%, on average across residents. The daily prevalences in Table 2 are likely to be conservative given incomplete shift registration (see Sensitivity to the number of registered shifts per day).

**Table 2:** Prevalence and severity distribution of individual BPSD symptoms

Symptom	Cumulative prevalence, %	Daily prevalence, %	Severity 1, %	Severity 2, %	Severity 3, %
Motor restlessness	84.8	17.6	7.8	6.6	3.2
Agitation	90.4	16.4	8.9	5.0	2.5
Anxiety	85.7	14.1	7.7	4.4	2.0
Irritability / emotional lability	86.1	13.5	7.9	4.0	1.6
Depression	87.0	13.3	7.3	4.2	1.8
Hallucinations	82.6	12.2	6.0	4.3	1.9
Appetite and eating disturbances	87.8	11.1	6.3	3.0	1.8
Delusions	83.5	11.1	5.8	3.4	1.8
Sleep disturbances	84.3	9.5	4.7	3.1	1.8
Disinhibition	76.1	6.5	3.4	2.2	1.0
Apathy	78.3	5.6	3.2	1.7	0.7
Euphoria / elation	70.4	4.3	2.5	1.2	0.6

Note. *N* = 230 residents. Symptoms are ordered by descending mean daily prevalence. Cumulative prevalence is the proportion of residents with at least one recorded occurrence of the symptom over the observation period. Daily prevalence is the percentage of registered days (days with at least one shift registration) on which the symptom was recorded. Daily prevalence and the severity columns are person-weighted means (each measure computed per resident, then averaged across the sample, giving each resident equal weight regardless of observation period length). The severity columns give the percentage of registered days on which the symptom was recorded at each level, based on the highest severity recorded that day. On the NPI-NH severity scale of 0 to 3, level 0 denotes absence and is not shown; the three severity columns therefore sum to the daily prevalence

**Fig. 3:**
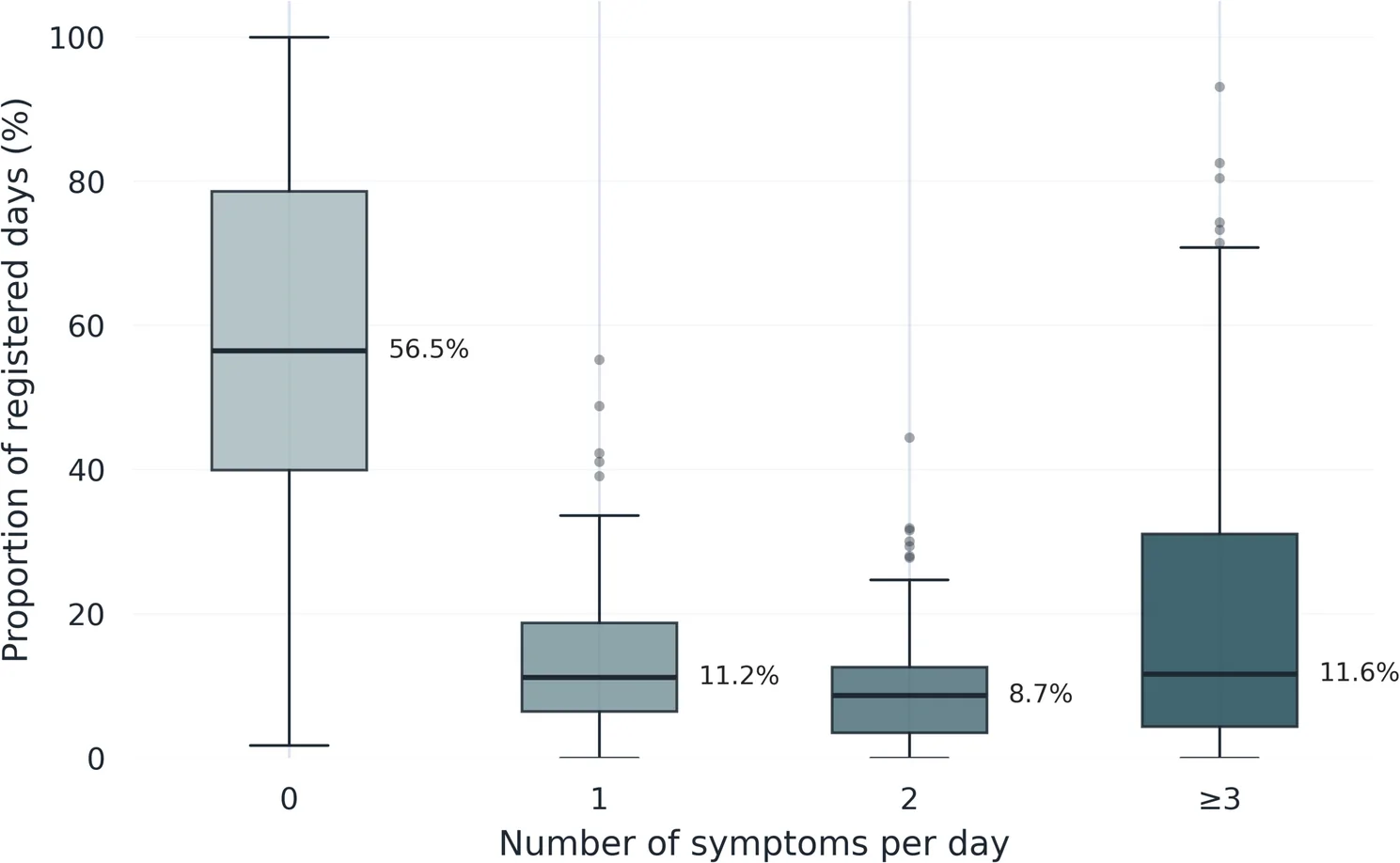
Distribution of daily BPSD symptom burden across individuals. Boxplots show the between-person distribution of the proportion of registered days (days with at least one shift registration) with 0, 1, 2, or ≥ 3 different NPI-NH symptoms recorded as present on the same day. Categories refer to the number of distinct symptoms (out of the twelve NPI-NH domains), not multiple recordings of the same symptom. Each box spans the interquartile range across individuals, the horizontal line marks the median (also annotated in percent beside each box), the whiskers extend to 1.5 times the interquartile range, and points beyond them are individual outliers

The distribution of daily symptom burden varied substantially between residents (Fig. 3). At least one symptom was recorded on a median of 43.5% of registered days, ranging from 0% to over 80% across residents, and 90 of 230 residents (39%) had at least one symptom recorded on more than half of their registered days. Days with exactly one symptom accounted for a median of 11.2% of registered days, and days with two symptoms for 8.7%. Days with three or more concurrent symptoms accounted for a median of 11.6% of individual registered days, but for some residents this proportion exceeded 80%. Symptom burden was unevenly distributed. The 20% of residents with the highest mean daily symptom count accounted for 49% of the total burden (Gini coefficient 0.48).

### Symptom co-occurrence

**Fig. 4:**
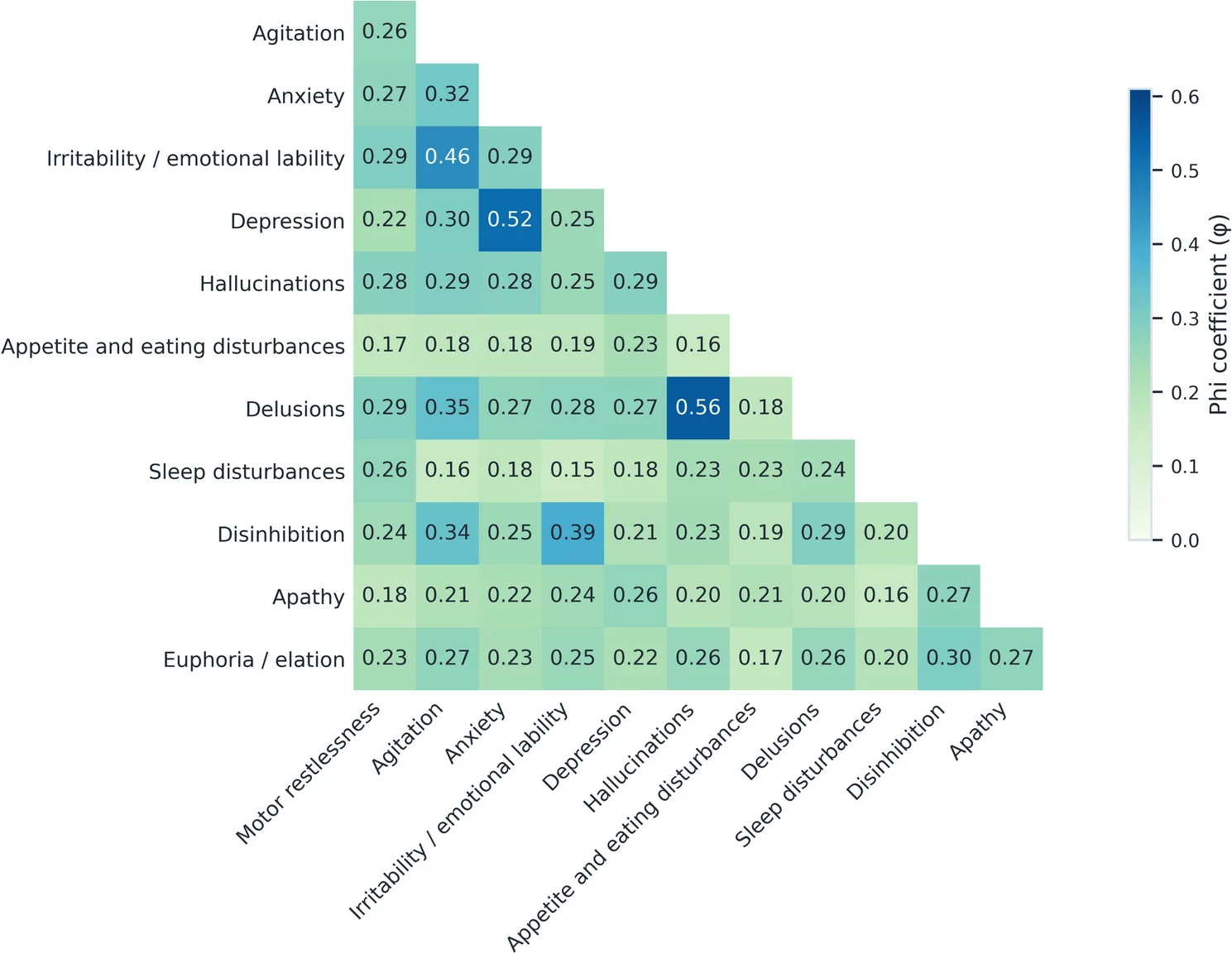
Person-weighted pairwise phi coefficients between BPSD symptoms. Each cell shows the mean phi coefficient for one symptom pair, computed separately for each individual from their binary day-level data and then averaged across individuals, with darker colors indicating stronger co-occurrence. Symptoms are ordered by descending mean daily prevalence, matching the ordering used in Table 2 andFig. 5. The phi coefficient quantifies the degree to which two symptoms co-occur beyond what would be expected from their individual prevalences

All 66 symptom pairs had positive phi coefficients, ranging from 0.15 to 0.56 (Fig. 4). Three pairs showed notably strong associations: delusions and hallucinations (φ = 0.56), anxiety and depression (φ = 0.52), and agitation and irritability (φ = 0.46). A second tier of moderate associations (φ = 0.34–0.39) involved disinhibition with irritability, agitation with delusions, and agitation with disinhibition. The remaining pairs had lower coefficients, all at or below φ = 0.32 (median across all 66 pairs 0.24).

### Between-unit variation

**Fig. 5:**
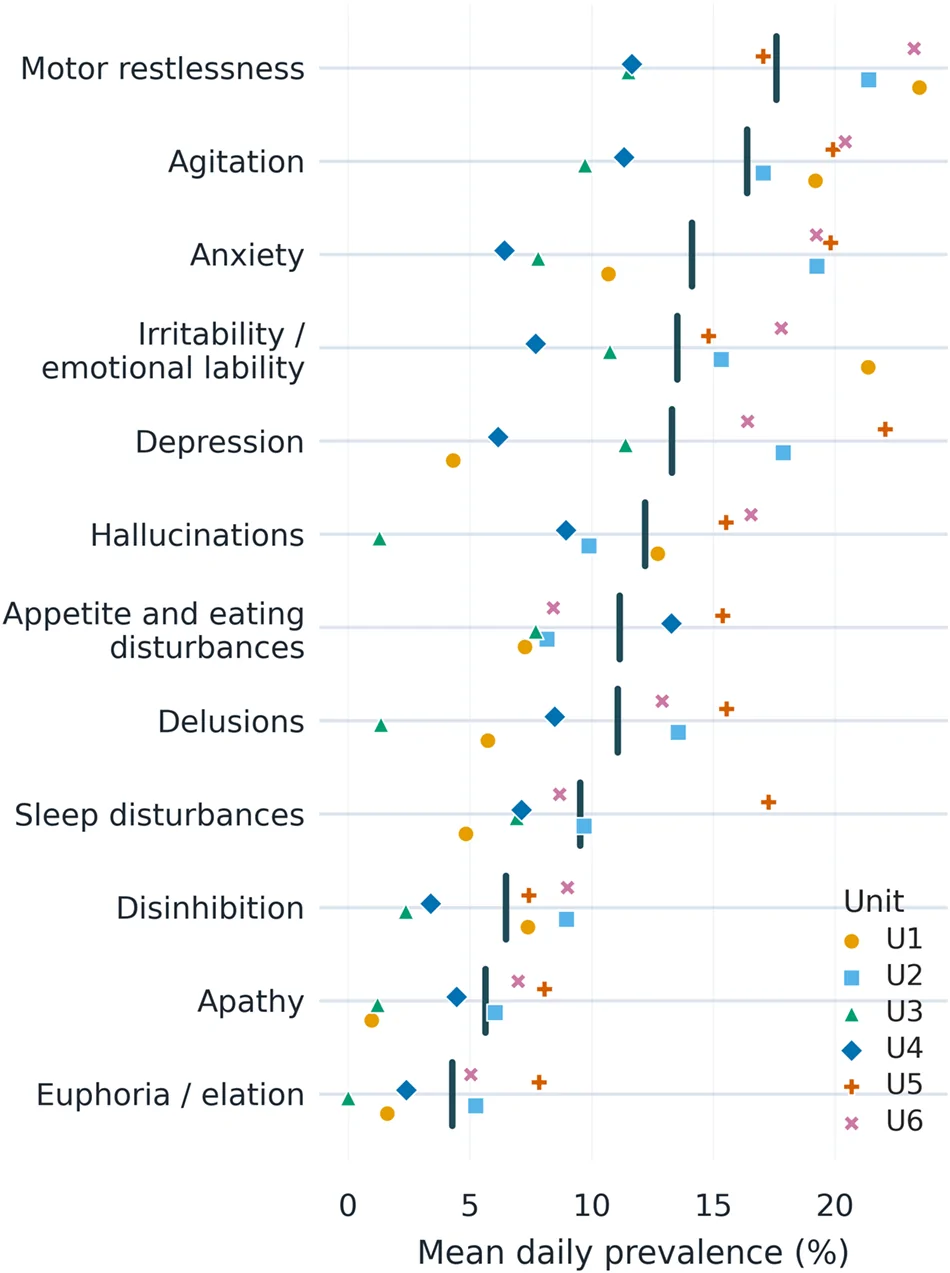
Between-unit variation in daily symptom prevalence. Each row represents one symptom, ordered by descending overall mean daily prevalence, matching the ordering used in Table 2 and Fig. 4. Dots indicate the person-weighted mean daily prevalence for each of the six care units, distinguished by color and marker shape (see legend). Vertical lines mark the overall mean across all individuals, giving each person equal weight

Mean daily symptom prevalence varied across the six care units for all twelve symptoms (Fig. 5). The mean number of symptoms recorded per person per day was highest in U5 (1.80) and U6 (1.62) and lowest in U3 (0.72) and U4 (0.90). As shown in Fig. 5, the mean daily prevalence of depression ranged from 4.3% (U1) to 22.1% (U5), and motor restlessness ranged from 11.5% (U3) to 23.5% (U1).

### Shift and weekday patterns

**Fig. 6:**
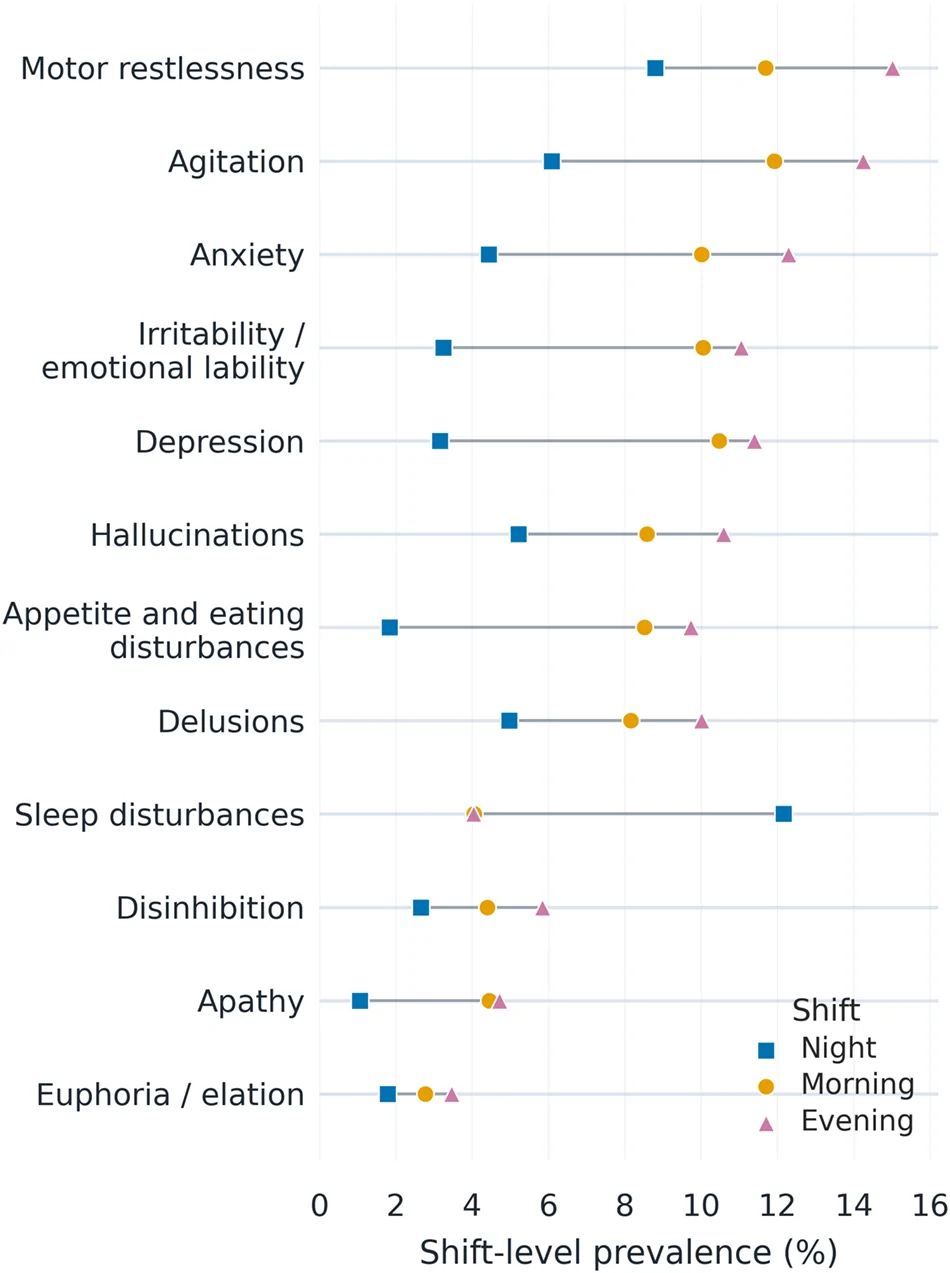
Shift-level prevalence of BPSD by shift. Each row represents one symptom, ordered by descending overall mean daily prevalence as in Table 2. Markers show the person-weighted proportion of shift registrations at each shift (night, morning, evening) in which the symptom was recorded, and the line spans the range across shifts. For sleep disturbances, the morning and evening values coincide (4.0%)

Symptoms were most often recorded during evening shifts and least often at night (Fig. 6). On average, 37.8% of evening, 33.6% of morning, and 18.4% of night shift registrations contained at least one symptom. The exception was sleep disturbances, which were recorded mainly at night (12.2% of night shift registrations versus 4.0% of morning and evening shift registrations). Variation across weekdays was small. The proportion of registered days with any symptom ranged from 40.4% (Sunday) to 43.9% (Monday), and the mean number of registered shifts per day was similar on all days (1.66–1.70).

### Sensitivity to the number of registered shifts per day

Because nearly half of the registered days rested on a single shift registration, the day-level results were repeated on days with all three shifts registered, and the co-occurrence analysis also at the level of single shift registrations (Additional file 1, Tables S4 and S5). Restricting the analyses to these days raised the levels. The daily prevalence of agitation, for example, was 23.0% on days with all three shifts registered compared with 16.3% on all registered days among the same residents, and the mean daily symptom count was 1.98 compared with 1.32. The patterns did not change. Within residents and within the same shift, shift registrations were about as likely to contain a symptom on days with one as with all three shifts registered, which is consistent with more opportunities to detect symptoms rather than selective documentation. The concentration of symptom burden, the co-occurrence structure, and the rank order of the units were similar on days with all three shifts, and phi computed from single shift registrations was practically identical to the day-level coefficients, although the differences between units were wider and the share of days with all three shifts varied widely between units.

## Discussion

This study describes BPSD as documented through daily registrations in six Swedish nursing homes. Drawing on over one million symptom registrations from 230 individuals followed for a median of 270 days, the findings show that cumulative symptom prevalence was high, while the daily symptom picture was predominantly mild and intermittent, with substantial variation both between individuals and between care units. Beyond replicating known patterns at higher temporal resolution, the study provides an empirical baseline of BPSD in routine nursing home care, in the form of a large-scale account at daily resolution with severity recorded per shift across all three shifts of the 24-hour cycle by the care staff who deliver everyday care.

### Symptom prevalence and severity in context

All twelve NPI-NH symptoms exceeded 70% cumulative prevalence over the median 270-day observation period, with agitation reaching 90.4% and depressive symptoms 87.0%. These cumulative figures are not directly comparable with cross-sectional point-prevalence estimates. For context, Seitz et al. [29], in a systematic review of long-term care populations, reported an overall BPSD prevalence of 78%, defined as the proportion of residents with dementia showing any symptom at assessment. At the individual symptom level, cross-sectional studies report substantially lower point prevalences. In the Swedish BPSD registry, with data on more than 10,000 residents, per-symptom prevalences of clinically significant symptoms ranged from 5.9% (euphoria) to 36.4% (motor restlessness), with agitation, motor restlessness, and irritability among the most frequently registered symptoms [3]. Similar ranges have been documented in Norwegian [30] and Dutch [31] nursing home cohorts. This discrepancy mainly reflects the difference between cumulative and point measures, as daily registrations accumulate observations over hundreds of days and capture symptoms that are infrequent or transient at the individual level. The gap also underscores the intermittent character of BPSD. While cumulative figures exceeded 70%, the mean proportion of registered days on which any given symptom was recorded ranged from only 4% to 18%. Period-based instruments such as the NPI-NH estimate whether a symptom was present during a recall period, typically the preceding month, and rely on staff recall across shifts they may not have worked. Daily shift-level registration instead records the number of days on which a symptom was documented. The two approaches answer different questions and are complementary. Because nearly half of the registered days rested on a single shift registration, the daily estimates are likely to be conservative, as shown by the higher prevalence on days with all three shifts registered.

Registered severity was predominantly mild across all twelve symptoms. Severity level 1 consistently accounted for the largest share of symptomatic days, while severity level 3 was rare, at most 3.2% of registered days (motor restlessness). Although infrequent, severity-3 episodes represent moments of greatest distress and elevated risk for the resident and those around them. Individual symptoms vary over time [32], and retrospective informant-based ratings are subject to recall bias [17]. Capturing severe episodes when they occur is therefore one of the principal motivations for continuous behavioral monitoring in this group. At the other end of the severity spectrum, previous research has shown that even symptoms that are infrequent or low in intensity can be associated with substantial caregiver distress, and that the relationship between symptom intensity and perceived burden varies across symptom domains [12]. The predominantly mild severity observed here may reflect a genuine characteristic of this population, or a measurement effect. When staff record symptoms as they occur rather than reconstructing them retrospectively, less severe episodes that might otherwise go unreported or be subsumed into a higher summary rating may be captured individually. The clinical significance of symptoms recorded frequently over extended periods remains an open question.

### Intermittency, between-person variation, and shift patterns

These findings align with evidence that BPSD fluctuate considerably over time. Van der Linde et al. [4] demonstrated that the persistence and incidence of individual symptoms vary substantially, with some symptoms, such as apathy, showing high persistence, and others, such as anxiety and depression, exhibiting more episodic patterns. Using an eight-day intensive longitudinal design, Pickering et al. [19] identified distinct within-person symptom factors that differed from between-person patterns, underscoring that the composition of BPSD varies not only across individuals but also within individuals over time. The present study adds a descriptive account at daily resolution over a considerably longer observation period. Within-person temporal dynamics were not analyzed and remain for future work.

The between-person variation in daily symptom burden was substantial. As shown above, the proportion of registered days with any symptom, and with three or more concurrent symptoms, varied widely between individuals. This heterogeneity is consistent with longitudinal studies showing that certain symptoms, particularly agitation, irritability, and apathy, are highly persistent in subgroups of nursing home residents, while affective symptoms follow a more fluctuating course [33]. Because a minority of individuals in this cohort accounted for a disproportionate share of the total symptom burden, the associated distress and care needs are likely to be similarly concentrated, which suggests that these individuals may warrant targeted monitoring and intervention.

Symptoms were most frequently recorded during evening shifts. Late-day worsening of behavioral symptoms, commonly described as sundowning, is a well-known clinical observation [34, 35], but direct observation in nursing homes has found agitation to peak in the mid-afternoon and decline thereafter, with a clear late-day increase in only about a quarter of residents [36]. The shift-level resolution of the present data cannot separate these descriptions, since the evening shift spans the afternoon and the evening, and the evening predominance is a group-level pattern that does not imply a late-day increase in every resident; within-person patterns were not examined. The predominance of sleep disturbances at night, alongside low levels of other symptoms, indicates that shift-level registration distinguishes symptom profiles that a single period-based rating would combine. No weekday pattern was observed, and the number of registered shifts per day was similar across weekdays, which suggests that the shift pattern reflects the time of day rather than the organization of the week.

### Symptom co-occurrence and between-unit variation

The three strongest co-occurring symptom pairs (delusions and hallucinations, anxiety and depression, and agitation and irritability) correspond to domains repeatedly identified in factor-analytic studies of the NPI and its nursing home version. Aalten et al. [37], pooling NPI data from multiple European memory-clinic cohorts, identified a psychosis factor (delusions, hallucinations), an affective factor (anxiety, depression), and a hyperactivity factor (agitation, irritability, motor restlessness) that closely mirror the clustering observed here. A similar and stable factor solution has been found in 1,437 Dutch nursing home patients [38]. That symptoms within these domains co-occur is expected given their conceptual proximity. What is informative is that the strongest pairwise associations at the day level mirror the structure observed in cross-sectional factor-analytic work, consistent with these symptom groupings holding across measurement approaches. At the same time, all 66 symptom pairs were positively associated (median φ = 0.24, range 0.15–0.56), also at the level of single shift registrations, consistent with a shared general symptom burden on which symptom-specific patterns are superimposed, although rater effects cannot be excluded, since all twelve domains are rated by the same staff member at the same shift. Daily monitoring may therefore benefit from both symptom-specific tracking and the daily number of symptoms.

The observed between-unit variation in symptom prevalence was substantial, consistent with findings from Zuidema et al. [22], who assessed 1,289 patients across 56 dementia special care units and found that 3.5–14.8% of the total variance in neuropsychiatric symptoms was attributable to differences between units, even after adjusting for patient-level factors. Several factors may contribute: differences in case mix (dementia diagnosis, disease stage, comorbidity), organizational factors (staffing levels, care routines, physical environment), and differences in how staff interpret and record symptoms. The tool relies on direct care staff to judge whether a behavior meets the threshold for a given symptom, and staff members’ training, experience, and familiarity with individual residents may lead to systematic differences in recording patterns between units. The present study, with only six units and incomplete individual-level diagnostic data, cannot disentangle these contributions. Doing so will require future analytical work, including multilevel decomposition of within-person, between-person, and between-unit variation; identification and characterization of residents with a high symptom burden; and formal factor-analytic replication of NPI-NH subsyndromes at the day level. The descriptive baseline established here provides a calibration point for such analyses.

### Implications for monitoring and care

These findings carry implications for how BPSD are monitored in routine nursing home care. Current practice in Sweden relies predominantly on periodic NPI-based assessments, typically conducted every six to twelve months [18], which are not designed to describe day-to-day variation. Daily registration shows how often each symptom is documented and on which shifts, how often severe ratings occur, and which symptoms are recorded together. The evening peak also raises the question of whether staffing levels and handover routines are aligned with the times when symptoms are most often recorded. Making that variation visible in routine practice could help care teams recognize residents whose symptom patterns warrant closer attention and tailor care to the specific picture each individual presents, both of which are commonly regarded as preconditions for person-centered dementia care [7, 8]. Care that responds to each person’s current symptom picture has the potential to reduce distress and better meet needs as they arise. Whether daily registration realizes these benefits in practice was not evaluated in the present descriptive study and is an important direction for future research. The application already shows each resident’s registrations over time to staff; adding summary measures such as those reported here, including the proportion of days with each symptom and the daily number of symptoms, at resident, ward, and unit level, could support care planning, quality follow-up, and evaluation of whether interventions are followed by changes in symptoms.

### Strengths and limitations

The study draws on a dataset of considerable scale and temporal density. All registrations were completed by direct care staff as part of their daily routines, rather than by trained research personnel, strengthening the ecological validity of the findings. The multi-site design, spanning six care units across six municipalities, captures variation in organizational context and care practices, and registrations cover all three shifts of the 24-hour cycle, including night shifts, which are rarely covered in existing BPSD research, although these were registered least often.

Several limitations should be noted. The study population was drawn from a convenience sample of six nursing homes, limiting generalizability. Comparable national data on unit characteristics were not available, so the representativeness of the participating units relative to other Swedish nursing homes could not be assessed. Two of the units (U1 and U3) had taken part in the pilot study. They were also small, with one ward each, and registered more shifts per registered day than any other unit (2.0 and 2.5, compared with 1.7 overall), which would, if anything, increase the number of recorded symptoms; nevertheless, both had mean daily symptom counts below the overall mean (Additional file 1, Table S2). Any effect of pilot participation on their symptom levels therefore cannot be separated from these differences. Dementia subtype and disease severity were neither uniformly recorded nor verified, so we could not examine whether BPSD patterns differ between subtypes, which differ in symptom patterns [3] and survival after diagnosis [39], or between stages of disease progression; nor were patterns examined by sex.

The active-use criterion (at least 15 days with registrations per 30-day block) and the trimming of each observation period may have excluded individuals with shorter stays or rapidly declining health. Excluded residents had fewer registered days and a lower proportion of symptomatic days than included residents, so excluding them is unlikely to have lowered the daily prevalence estimates, although their few registered days make their true symptom level uncertain. Because data on death or discharge were not available, we could not assess whether residents with severe symptoms and short survival were under-represented. Observation period also varied substantially between individuals (median 270 days, range 24 to 719), which may influence both cumulative prevalence estimates (which favor individuals followed for longer) and the apparent between-person variation in symptom burden. Registration completeness varied across units (approximately 71% to 99% of observation days; Fig. 2) and was lower during night shifts and summer months, suggesting that organizational factors influenced how consistently registrations were made. Units differed in the number of shifts registered per registered day (1.4 to 2.5), which could in itself produce between-unit differences; restricting the analyses to days with all three shifts raised the levels but left the between-unit pattern and the co-occurrence structure broadly similar. The within-resident comparison of days with one and with all three shifts registered does not suggest that registration depended strongly on symptom occurrence.

Registrations were carried out by staff with varying levels of experience, and the interpretation of observed behavior involves a degree of subjectivity. Inter-rater variability is inherent to observational data collected in routine care and distinguishes the present dataset from designs employing trained raters with established reliability [40]. Inter-rater reliability of the tool has not been assessed, and the pilot [21] evaluated feasibility and integration into routine care rather than psychometric properties. The Daily-BPSD tool uses the twelve NPI-NH symptom domains and its severity scale, adapted for registration at each shift. Its validity against standard NPI-NH administration has not been established, and the prevalences reported here should therefore be interpreted as documented symptom occurrence rather than as validated clinical assessments. Because the tool captures severity but not frequency as a separate dimension, symptom burden may also be characterized differently than in studies using standard NPI-NH scoring. The non-objection procedure was carried out locally by the unit managers, and the research team was therefore not in a position to verify that it had been completed for each individual resident. The data exhibit a nested hierarchical structure (registrations within shifts, days, residents, wards, and units) where observations within the same cluster are not independent. While this does not introduce bias in descriptive statistics, any future inferential analyses must account for these dependencies [41].

## Conclusions

Daily registrations offer a picture of BPSD at a temporal resolution that periodic assessments are not designed to provide. Although cumulative prevalence was high across all twelve NPI-NH symptoms, the daily symptom picture was predominantly mild and intermittent, with at least one symptom recorded on a median of 43.5% of registered days, a conservative estimate given incomplete shift registration. At the same time, substantial heterogeneity was observed. Symptom burden varied widely between individuals, and 90 of 230 residents (39%) had at least one symptom recorded on more than half of their registered days. Symptom co-occurrence followed established NPI-NH domain structures, prevalence varied considerably across care units, and symptoms were recorded most often on evening shifts. Such registration shows how often each symptom is documented and on which shifts, and complements period-based assessments rather than replacing them. The descriptive baseline established here provides a foundation for future multilevel analyses and may support the development of more responsive, individualized approaches to BPSD monitoring in nursing homes.

## Supplementary information

Supplementary Material 1

## Data Availability

The data that support the findings of this study are not publicly available. Data were collected through the Daily-BPSD tool in clinical nursing home settings and contain sensitive personal health information. Access may be requested from the corresponding author, subject to applicable data protection legislation and ethical approval.

## Declarations

### Generative AI and AI-assisted technologies in the manuscript preparation process

During the preparation of this work the authors used Claude (Anthropic) in order to support code optimization, visualization, proofreading and editing, reformatting, and publication support, under full human supervision, according to the GAIDeT taxonomy. All study design, data collection, analysis, interpretation, and conclusions were carried out by the authors, who reviewed and edited all AI-assisted output and take full responsibility for the content of the published article.

## Abbreviations

BPSD: Behavioral and psychological symptoms of dementia
GDPR: General Data Protection Regulation
IQR: Interquartile range
NPI-NH: Neuropsychiatric Inventory, Nursing Home version
PRN: Pro re nata (as-needed medication)
STROBE: Strengthening the Reporting of Observational Studies in Epidemiology
