# Supplementary material for: Daily patterns and prevalence of behavioral and psychological symptoms of dementia in Swedish nursing homes
Source: BMC Geriatr. 2026 Oct 29;26:1415. doi: 10.1186/s12877-026-08537-w (PMC13757153; doi:10.1186/s12877-026-08537-w)
Supplement: Supplementary file 1 [file 12877_2026_8537_MOESM1_ESM.docx]

Additional file 1: Supplementary Tables S1–S5

Daily Patterns and Prevalence of Behavioral and Psychological Symptoms of Dementia in Swedish Nursing Homes

## Supplementary Table S1. STROBE checklist

Checklist of items that should be included in reports of observational studies, following the STROBE Statement [1]. This study is a descriptive longitudinal observational study.

| Item | STROBE recommendation | Reported | Location |
| --- | --- | --- | --- |
|  | **Title and abstract** |  |  |
| 1 | Indicate the study’s design with a commonly used term in the title or abstract | Yes | Abstract (Methods) |
|  | **Introduction** |  |  |
| 2 | Explain the scientific background and rationale for the study | Yes | Background, paragraphs 1–6 |
| 3 | State specific objectives, including any prespecified hypotheses | Yes | Background, final paragraph (aims) |
|  | **Methods** |  |  |
| 4 | Present key elements of study design early in the paper | Yes | Methods, opening paragraph |
| 5 | Describe the setting, locations, and relevant dates | Yes | Setting and data source (collection period January 2024–December 2025; units started January–October 2024); Supplementary Table S2 (start date per unit) |
| 6 | Give the eligibility criteria, and the sources and methods of selection of participants | Yes | Setting and data source (recruitment of units); Study population (all residents with registrations considered; active-use criterion); Ethics and data access (non-objection procedure) |
| 7 | Clearly define all outcomes, exposures, predictors, potential confounders, and effect modifiers | Yes | Variables and data structure |
| 8 | For each variable of interest, give sources of data and details of methods of assessment | Yes | Setting and data source; Variables and data structure |
| 9 | Describe any efforts to address potential sources of bias | Yes | Statistical approach (person-weighting; daily prevalence also on days with all three shifts registered, Supplementary Table S4; within-resident comparison of days with one and three shifts; between-unit and co-occurrence analyses repeated on days with all three shifts); Results (Sensitivity to the number of registered shifts per day; Supplementary Table S5); Strengths and limitations |
| 10 | Explain how the study size was arrived at | Yes | Study population (active-use criterion); Figure 1; Supplementary Table S3 (sensitivity analysis of the criterion) |
| 11 | Explain how quantitative variables were handled in the analyses | Yes | Variables and data structure; Statistical approach |
| 12 | Describe all statistical methods, including how missing data were addressed | Yes | Statistical approach (observed registrations only; registered days as denominator; definitions of completeness; sensitivity analyses); Results (Study population and data completeness; Sensitivity to the number of registered shifts per day); Strengths and limitations (registration and symptom occurrence) |
|  | **Results** |  |  |
| 13 | Report numbers of individuals at each stage of study and reasons for non-participation | Yes | Study population; Figure 1 (flowchart, including excluded residents per unit); Supplementary Table S2; residents never registered cannot be counted (occupancy data not available; Results); Ethics and data access (objections handled locally by unit managers and not reported to the research team) |
| 14 | Give characteristics of study participants and information on exposures and potential confounders | Yes | Table 1 (sex and age available for all 230 residents); Supplementary Table S2 (units); Results (excluded compared with included residents); individual-level diagnostic detail not analyzed (Strengths and limitations) |
| 15 | Report numbers of outcome events or summary measures | Yes | Results (Table 2, Figures 3–6); Supplementary Table S4 |
| 16 | Give unadjusted estimates and, if applicable, confounder-adjusted estimates | Yes (unadjusted only) | Table 2, Figures 3–6; no adjusted estimates (descriptive design) |
| 17 | Report other analyses done | Yes | Results (completeness, co-occurrence, between-unit variation, shift and weekday patterns, sensitivity to the number of registered shifts per day); Supplementary Tables S4 and S5 |
|  | **Discussion** |  |  |
| 18 | Summarize key results with reference to study objectives | Yes | Discussion, opening paragraph |
| 19 | Discuss limitations of the study, taking into account sources of potential bias | Yes | Strengths and limitations |
| 20 | Give a cautious overall interpretation considering objectives, limitations, multiplicity of analyses | Yes | Discussion; Conclusions |
| 21 | Discuss the generalizability of the study results | Yes | Strengths and limitations |
|  | **Other information** |  |  |
| 22 | Give the source of funding and the role of the funders | Yes | Declarations, Funding |

## Supplementary Table S2. Characteristics of the participating units

| Unit | Ownership | Wards | Start | End | Res. | Excl. | Incl. | Obs. days | Compl., % | Shifts per day | 3-shift days, % | Sympt./day |
| --- | --- | --- | --- | --- | --- | --- | --- | --- | --- | --- | --- | --- |
| U1 (pilot) | Municipal | 1 | Jan 2024 | Dec 2025 | 14 | 1 | 13 | 319 | 97.9 | 2.04 | 25.9 | 1.19 |
| U2 | Municipal | 2 | Mar 2024 | Aug 2025 | 32 | 5 | 27 | 319 | 83.5 | 1.72 | 16.4 | 1.51 |
| U3 (pilot) | Municipal | 1 | Feb 2024 | Oct 2024 | 10 | 0 | 10 | 60 | 98.6 | 2.50 | 56.2 | 0.72 |
| U4 | Municipal | 6 | Feb 2024 | Dec 2025 | 83 | 7 | 76 | 298 | 78.9 | 1.60 | 11.5 | 0.90 |
| U5 | Private | 3 | Oct 2024 | Dec 2025 | 46 | 5 | 41 | 330 | 89.0 | 1.96 | 26.6 | 1.80 |
| U6 | Private | 5 | Sep 2024 | Dec 2025 | 83 | 20 | 63 | 239 | 71.1 | 1.39 | 2.0 | 1.62 |
| All | 4 municipal, 2 private | 18 | Jan 2024 | Dec 2025 | 268 | 38 | 230 | 270 | 81.0 | 1.68 | 14.9 | 1.34 |

*Note.* Units are nursing homes; all wards were specialized dementia care wards. Start and End are the months of the first and last registration among the included residents in the unit. Res. are all residents with at least one Daily-BPSD registration; Excl. and Incl. are the numbers excluded (not meeting the active-use criterion in any 30-day period) and included. Obs. days is the median observation period in days; Compl. (day-level completeness), Shifts per day (shifts per registered day), 3-shift days (share of registered days with all three shifts), and Sympt./day (symptoms per registered day) are person-weighted means; all refer to the included residents. Day-level completeness is the proportion of observation days with at least one shift registered; shifts per registered day is the mean number of shifts (1 to 3) registered on registered days, which is what governs how many symptoms can be recorded. The All row gives the totals, the median observation period, and the person-weighted means for all 230 included residents. Wards are those with included residents; three further wards had only excluded residents. U1 and U3 had taken part in the pilot of the tool.

## Supplementary Table S3. Sensitivity analysis of the active-use criterion

The study applied an active-use criterion requiring at least 15 registered days per 30-day block. To assess the sensitivity of this threshold, Table S3 presents the effect of three thresholds (10, 15, and 20 days) on the number of included residents, total item-level registrations, and person-weighted day-level completeness. Each threshold was applied as the sole criterion using the same active-use procedure described in the Methods, varying only the required number of registered days per 30-day block.

| Threshold | Residents remaining | Item-level registrations remaining | Day-level completeness, % |
| --- | --- | --- | --- |
| 10 days (1 in 3) | 246 (92%) | 1,105,438 (98%) | 75.9 |
| 15 days (1 in 2) | 230 (86%) | 1,065,312 (94%) | 81.0 |
| 20 days (2 in 3) | 215 (80%) | 996,334 (88%) | 85.7 |

*Note.* Percentages in parentheses refer to the proportion of the original 268 residents and 1,131,706 item-level registrations retained at each threshold. The threshold of 15 days (registrations on at least every other day) was selected as the primary criterion, balancing sample size against data quality. Moving from 10 to 15 days excluded 16 additional residents while increasing completeness by 5.1 percentage points. Moving from 15 to 20 days excluded 15 further residents for a 4.6 percentage point gain in completeness.

## Supplementary Table S4. Daily prevalence by number of registered shifts and shift-level prevalence

| Symptom | All days, % | 3-shift days, % | Night, % | Morning, % | Evening, % |
| --- | --- | --- | --- | --- | --- |
| Motor restlessness | 17.3 | 24.3 | 8.8 | 11.7 | 15.0 |
| Agitation | 16.3 | 23.0 | 6.1 | 11.9 | 14.2 |
| Anxiety | 13.4 | 19.0 | 4.4 | 10.0 | 12.3 |
| Irritability / emotional lability | 13.5 | 19.0 | 3.2 | 10.1 | 11.1 |
| Depression | 12.2 | 16.9 | 3.2 | 10.5 | 11.4 |
| Hallucinations | 11.8 | 17.9 | 5.2 | 8.6 | 10.6 |
| Appetite and eating disturbances | 10.9 | 18.7 | 1.8 | 8.5 | 9.7 |
| Delusions | 11.5 | 16.2 | 5.0 | 8.2 | 10.0 |
| Sleep disturbances | 9.9 | 17.3 | 12.2 | 4.0 | 4.0 |
| Disinhibition | 6.6 | 8.4 | 2.7 | 4.4 | 5.8 |
| Apathy | 5.7 | 9.7 | 1.1 | 4.4 | 4.7 |
| Euphoria / elation | 4.3 | 7.0 | 1.8 | 2.8 | 3.5 |
| Any symptom | 41.6 | 56.5 | 18.4 | 33.6 | 37.8 |

*Note.* All values are person-weighted percentages. Symptoms are ordered as in Table 2. The first two columns are computed for the same residents: the 185 of 230 residents who had at least one day on which the night, morning, and evening shifts were all registered. All days is daily prevalence on all registered days (days with at least one shift registration) for these residents; the corresponding values for all 230 residents are given in Table 2. 3-shift days is the same measure restricted to days on which all three shifts were registered. Night, morning, and evening give the proportion of shift registrations at that shift in which the symptom was recorded, for all 230 residents. The row Any symptom gives the proportion of days (first two columns) or shift registrations (last three columns) with at least one symptom. Of the registered days, 53.5% had at least two shifts and 14.9% all three. For the 185 residents, the mean number of symptoms per day was 1.98 on days with all three shifts and 1.32 on all their registered days.

## Supplementary Table S5. Sensitivity of day-level results to the number of registered shifts per day

**A. Shift registrations with at least one symptom on days with one and with all three shifts registered, compared within residents and within the same shift**

| Shift | Residents with both types of day | Days with one shift, % | Days with all three shifts, % | Mean within-resident difference, percentage points |
| --- | --- | --- | --- | --- |
| Night | 173 | 16.9 | 17.6 | +0.7 |
| Morning | 180 | 32.6 | 31.2 | –1.5 |
| Evening | 178 | 34.9 | 38.7 | +3.8 |

**B. Concentration of daily symptom burden between residents**

| Basis | Residents | Gini coefficient | Share of total burden, top 20% of residents, % |
| --- | --- | --- | --- |
| All registered days | 230 | 0.48 | 49.4 |
| All registered days, residents with at least one day with all three shifts | 185 | 0.47 | 48.9 |
| Days with all three shifts | 185 | 0.48 | 49.8 |

**C. Symptom co-occurrence (phi) by unit of analysis**

| Basis | Pairs with positive phi | Median phi | Range | Residents contributing per pair, minimum (median) | Rank correlation with day-level coefficients |
| --- | --- | --- | --- | --- | --- |
| All registered days (as in Figure 4) | 66 of 66 | 0.24 | 0.15–0.56 | 147 (177) | (reference) |
| Days with all three shifts | 66 of 66 | 0.26 | 0.14–0.60 | 77 (105) | 0.87 |
| Single shift registrations | 66 of 66 | 0.24 | 0.13–0.56 | 147 (177) | 0.99 |

**D. Mean daily symptom count per unit on all registered days and on days with all three shifts**

| Unit | Residents, all days | Residents, days with all three shifts | Days with all three shifts, % | Symptoms per day, all days (rank) | Symptoms per day, days with all three shifts (rank) |
| --- | --- | --- | --- | --- | --- |
| U1 | 13 | 13 | 25.9 | 1.19 (4) | 1.41 (5) |
| U2 | 27 | 24 | 16.4 | 1.51 (3) | 2.14 (3) |
| U3 | 10 | 10 | 56.2 | 0.72 (6) | 0.84 (6) |
| U4 | 76 | 65 | 11.5 | 0.90 (5) | 1.44 (4) |
| U5 | 41 | 41 | 26.6 | 1.80 (1) | 2.60 (2) |
| U6 | 63 | 32 | 2.0 | 1.62 (2) | 2.72 (1) |

*Note.* All percentages, means, and coefficients are person-weighted. Part A compares, for each resident and each shift, the proportion of that shift’s registrations containing at least one symptom on days with exactly one registered shift and on days with all three, among residents who had both types of day; the last column is the mean of the within-resident differences. Part B: total burden is the sum of residents’ mean daily symptom counts; the Spearman rank correlation between residents’ mean number of shifts per registered day and their mean daily symptom count was 0.18 (n = 230, reported descriptively). Part C: phi was computed per resident and averaged, as in Figure 4; on days with all three shifts fewer residents showed variation in both symptoms of a pair; the rank correlation is Spearman’s correlation between the 66 coefficients and those computed on all registered days. Part D: restricted to the residents with at least one day with all three shifts, the all-days means were 1.19, 1.59, 0.72, 0.86, 1.80, and 1.69 for U1 to U6, with the same rank order. The unit with the lowest daily prevalence was the same on both bases for 11 of 12 symptoms, and the rank order of units was similar for every symptom (Spearman rank correlation between the two bases 0.77 to 1.00).

1. von Elm E, Altman DG, Egger M, Pocock SJ, Gøtzsche PC, Vandenbroucke JP. The Strengthening the Reporting of Observational Studies in Epidemiology (STROBE) statement: Guidelines for reporting observational studies. Lancet. 2007;370:1453–7. <https://doi.org/10.1016/S0140-6736(07)61602-X>.
